# A randomized crossover study of the effect of butorphanol–lidocaine and tramadol–lidocaine on sevoflurane's minimum alveolar concentration in dogs

**DOI:** 10.3389/fvets.2022.1057580

**Published:** 2023-01-12

**Authors:** Mohamed Marzok, Adel I. Almubarak, Mahmoud Kandeel, Wael El-Deeb, Hussein Babiker, Sayed Fathi El-Hawari

**Affiliations:** ^1^Department of Clinical Sciences, College of Veterinary Medicine, King Faisal University, Al-Ahsa, Saudi Arabia; ^2^Department of Surgery, Faculty of Veterinary Medicine, Kafrelsheikh University, Kafrelsheikh, Egypt; ^3^Department of Pharmacology, Faculty of Veterinary Medicine, Kafrelsheikh University, Kafrelsheikh, Egypt; ^4^Department of Biomedical Sciences, College of Veterinary Medicine, King Faisal University, Al-Ahsa, Saudi Arabia; ^5^Department of Internal Medicine, Infectious Diseases and Fish Diseases, Faculty of Veterinary Medicine, Mansoura University, Mansoura, Egypt; ^6^Department of Surgery, Anesthesiology and Radiology, Faculty of Veterinary Medicine, Sohag University, Sohag, Egypt

**Keywords:** butorphanol, dog, minimum alveolar concentration, sevoflurane, tramadol

## Abstract

Inhalational anesthesia is routinely used in small animal surgery. Selecting a suitable drug combination is vital since it may negatively affect the patient's physiological condition. We conducted this study to examine the sparing effect of butorphanol–lidocaine (BUT–LID) and tramadol–lidocaine (TRM–LID) on sevoflurane's minimum alveolar concentration (MAC) in 10 healthy mongrel dogs aged 1–2 years and weighing 11.5 ± 0.8 kg (mean ± SD). Sevoflurane's MAC was measured on three separate occasions. The three dog treatment groups were control (CONT) anesthetized only with sevoflurane, TRM–LID (TRM, i.v. 1.5 mg kg^−1^, then 1.3 mg kg^−1^ h^−1^ and LID, i.v. 2 mg kg^−1^, then 3 mg kg^−1^ h^−1^) or BUT–LID treatment (BUT, i.v. 0.1 mg kg^−1^ then 0.2 mg kg^−1^ h^−1^ and LID, i.v. 2 mg kg^−1^, then 3 mg kg^−1^ h^−1^). We hypothesized that both TRM–LID and BUT–LID would result in a significant MAC sparing effect in healthy dogs. The TRM–LID treatment resulted in a non-significant MAC reduction. MAC was lowered significantly in the BUT–LID group (*p* = 0.009). The sevoflurane MAC-sparing effects of TRM–LID and BUT–LID treatments were 7.05 ± 22.20 and 19.90 ± 5.91%, respectively, a difference that was not statistically significant (*p* = 0.13). Bradycardia was observed in the TRM–LID (*p* < 0.001) treatment. The esophageal temperature was significantly higher for the TRM–LID treatment than the CONT (*p* < 0.001) treatment. No statistically significant changes were detected between the three groups in *f*_R_, Pe′CO_2_, and MABP. In conclusion, there was a significant sparing effect after adding BUT–LID co-infusion than the control group. No sparing effect was noticed when adding TRM–LID co-infusion. However, no difference in the MAC sparing percentages between the TRM–LID and BUT–LID treatments. The BUT–LID co-infusion resulted in a sevoflurane MAC reduction superior to TRM–LID in addition to minimal cardiorespiratory changes. Both BUT-LID and TRM-LID may be clinically beneficial to dogs during anesthesia. However, BUT-LID produced higher sparing effect and reduction of sevoflurane MAC value.

## Introduction

The minimum alveolar concentration (MAC) is the partial pressure of a gas that immobilizes 50% of individuals subjected to a noxious, supramaximal stimulus. MAC measurements are used to evaluate the potency of an inhaled anesthetic (1). MAC are related to the sedative, muscular relaxation, and analgesic effects of drugs and are a useful marker in evaluating how various drugs demonstrate a sparing effect on anesthetics that are inhaled.

Sevoflurane is a widely used volatile anesthetic drug (2, 3) with a low blood/gas partition coefficient that induces anesthesia and recovery rapidly (4). It may result in dose-dependent complications; in dogs, these complications include hypotension and impaired cardiac contractility (5, 6). Therefore, it is important to decrease the amount of inhalational anesthetic agents used and undesirable cardiorespiratory effects in sevoflurane anesthesia.

Tramadol has analgesic properties due to its mild μ-receptor agonist action and the inhibition of serotonin and norepinephrine uptake in the spinal cord pain pathways (7, 8). Administration of tramadol to dogs as one i.v. dose at a constant rate infusion (CRI) may reduce the required percent of inhaled anesthetics (9–11). Lidocaine, which blocks sodium channels, is i.v. administered for analgesia during inhalation of anesthesia. In dogs, it decreases the required doses of isoflurane (12, 13) and sevoflurane (14, 15). Butorphanol induces mild analgesia through its action as an agonist at κ-receptors and an antagonist at μ-receptors (16). Butorphanol administered i.v. may serve as a perioperative analgesic in dogs (17–19).

Previous studies in dogs have investigated how multimodal analgesics may provide a sparing effect for inhaled anesthetics (1, 20–23). However, to the best of our knowledge, no studies have compared the sparing effects of tramadol–lidocaine (TRM–LID) and butorphanol–lidocaine (BUT–LID) CRI in sevoflurane-anesthetized dogs. The main goal of this study was to evaluate TRM–LID and BUT–LID MAC sparing effect during sevoflurane use in dogs. We also determined the cardiorespiratory parameter changes during anesthesia for each treatment. The present study hypothesized that co-infusing TRM-LID and BUT-LID would reduce the amount of sevoflurane required in healthy dogs.

## Materials and methods

### Study design

This randomized crossover study included 10 female mongrel dogs aged 1–2 years and weighing 11.5 ± 0.8 kg (mean ± SD). Food withheld for 12 h and water for 1 h before the experiment. The results of physical examination, blood biochemical examination and complete blood counts indicated that all dogs were healthy. Each dog received one of three treatments on three occasions with 10-day wash-out periods. Allocations of dogs to groups was performed through online free software program (Random Allocation Software). We blindly determined the MAC for each dog. M.M. was the person responsible for MAC determination and he didn't know the infused analgesic drug used in each occasion. The Animal Care Committee of King Faisal University (approval no. KFUREC–ETHICS 59) reviewed and approved this protocol in correspondence with Saudi Arabianethical codes for studies on experimental animals.

### Anesthetic monitoring

Respiratory gas analyzer was calibrated for sevoflurane before each experiment. Inhaled sevofluorane was used to induce anesthesia (Sojourn, Julphar Co., Ltd., KSA) in 100% oxygen provided by face-mask and orotracheal intubation. After induction, we maintained anesthesia at 2.5% (Fe′Sevo) while the dogs were left laterally recumbent. Mechanical ventilation that was volume-controlled was initiated with a tidal volume of 15 ml kg^−1^ and a respiratory rate (*f*_R_) of 12 breaths minute^−1^ using a time-cycled ventilator (Nuffield Anesthesia Ventilator Series 200, Penlon, UK). The end-tidal carbon dioxide pressure (Pe′CO_2_) was maintained at 35–40 mm Hg *via* tidal volume adjustment. The esophageal temperature (T) was measured by using an oral electronic thermometer probe within the thoracic esophagus. The temperature was maintained at 37.5–38.5°C using a warm air blanket. The following parameters [electrocardiography (ECG lead 2), T, heart rate (HR), *f*_R_, oxygen saturation (SpO_2_), mean arterial pressure (MAP) measured oscillometrically, Pe′CO_2_ and Fe′Sevo.] were measured at 5min intervals using a patient monitoring apparatus (BM7 VET; Bionet, Republic of Korea). Side-stream capnography and an anesthetic agent monitor were used to measure Pe′CO_2_ and Fe′Sevo.

### MAC assessment

The sevoflurane MAC was measured *via* the tail clamp method, described previously (20–23). Towel clamp was used to induce pressure for 1 min around the mid-tail. Positive result means substantial movement of the head, neck, fore and hindlimbs of the dog. In case of positive results, the Fe′Sevo was increased by 10%, while in negative results the Fe′Sevo was decreased by 10%. Dog was tested again after 20 min of sevoflurane re-equilibration. Sevoflurane MAC was calculated as the mean of the Fe′Sevo when the dog showed two successive negative and positive responses. We calculated the mean MAC for each dog from triplicate measurements. We also recorded the time elapsed from the beginning of lateral recumbency until the end of triplicate MAC measurements. Cardiorespiratory variables were measured (T, HR, MAP, SpO_2_, Pe′CO_2_, *f*_R_) every 5 and 2 min before applying of a stimulus.

### TRM–LID and BUT–LID treatment experiments

The MAC of sevoflurane for each dog was measured whenever they received the control treatment (CONT; lactated Ringer's solution, Ringer lactate, Pharmaceutical Solution Industry, Jeddah, KSA) administered at 5 ml kg^−1^ h^−1^; TRM–LID (Tramal Injection, Memphis Co., Ltd., Cairo, Egypt) administered at a 1.5 mg kg^−1^ loading dose, then 1.3 mg kg^−1^ h^−1^, and lidocaine (xylocaine 2% for i.v. injection, Astra Zeneca Pharma India Ltd) loading dose administered at 2 mg kg^−1^, then 3 mg kg^−1^ h^−1^ CRI; BUT–LID (Torbugesic Vet, Zoetis, Belgium) BUT administered at 0.1 mg kg^−1^ i.v., then 0.2 mg kg^−1^ h^−1^ and lidocaine, 2 mg kg^−1^ i.v., then 3 mg kg^−1^ h^−1^). We administered the loading doses of all analgesics over 1 min intravenously. For CRI, we added each analgesic to lactated Ringer's solution, then administered at 5 ml kg^−1^ h^−1^.

Each dog's MAC sparing rate was calculated using the following equation: sevoflurane MAC-sparing rate (%) = MAC (CONT)-MAC (treatment) / MAC (CONT) × 100%.

### Statistical analysis

IBM SPSS 22 software program was used for statistical analyses. We used the Shapiro-Wilk test to analyse data for normality. HR, MAP, T, total time of anesthesia and MAC variables follow normal distribution. While Pe′CO_2_ and *f*_R_ SpO2 don't follow normal distribution so they were transformed to be normal. Sparing ratio was analyzed by non-parametric related sample test (Friedmans ANOVA test followed by Wilcoxon Signed Ranks test). We analyzed differences in MAC values and all cardiorespiratory variables *via* a repeated-measures one-way analysis of variance followed by a *post-hoc* test that adjusted *p*-values using a Bonferroni correction. All data were expressed as mean ± standard deviation. A *p* < 0.05 was statistically significant.

## Results

No dogs suffered any complications during the experiment. The study was conducted over 181 ± 33, 224 ± 50, and 193 ± 35 min for CONT, TRM–LID and BUT–LID treatment groups, respectively. The time required for MAC determination for TRM–LID was significantly longer than for CONT (*p* = 0.028) and for BUT–LID (*p* = 0.015) treatments.

**Figure 1:**
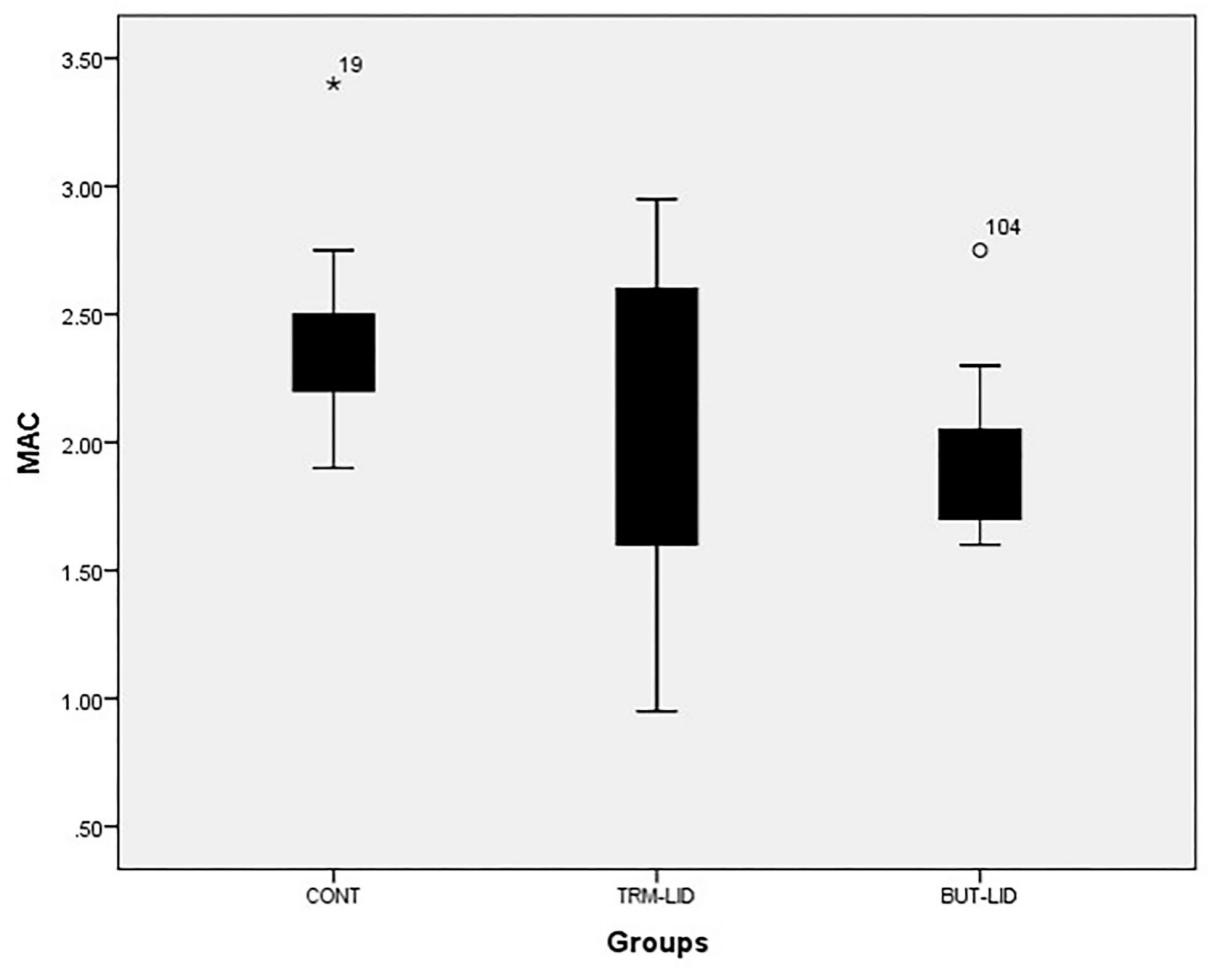
Mean values of minimum alveolar concentration (MAC) of sevoflurane in control (CONT), tramadol-lidocaine (TRM-LID) and butrophanol-lidocaine (BUT-LID) treatments in dogs.

The sevoflurane MAC sparing ratio was significantly lower in the BUT–LID group (*p* = 0.005) than in the CONT group. There were no statistically significant differences between CONT and TRM–LID (*p* = 0.33) or between TRM–LID and BUT–LID (*p* = 0.157) treatments (Table 1). However, significant reduction in MAC value was detected in BUT–LID group (*p* = 0.01) than CONT group and there was no significant variation of MAC value between TRM–LID and CONT treatments (*p* = 0.281). There was no significant difference in MAC values between TRM–LID and BUT–LID treatments (Figure 1).

**Table 1:** Mean ± SD MAC values (%) and degree of sparing effect (%) in control (CONT), tramadol-lidocaine (TRM-LID), butorphanol-lidocaine (BUT-LID) treatments obtained from 10 female dogs in each treatment.

	**MAC value (%)**	**Sparing effect (%)**
**CONT**	2.4 ± 0.36^‡^	N/A
**TRM-LID**	2.1 ± 0.53	7.05 ± 22.20
**BUT-LID**	1.9 ± 0.29^*^	19.90 ± 5.91^*^

^*^Statistically different from the CONT (p < 0.05). ^‡^Statistically different from the BUT-LID (p < 0.05). N/A, Not Available.

The cardiorespiratory variables measured 2 min before applying the noxious stimulus to the dogs in each treatment showed that the HRs were significantly lower in the TRM–LID than in the CONT group (*p* < 0.001). The T values were significantly higher in the TRM–LID treatment than in CONT (*p* < 0.001). Slight hypoexemia was noticed in TRM–LID treatment than in CONT (*p* = 0.003). No statistically significant changes were detected between the three groups for *f*_R_, Pe′CO_2_ and MABP. Also, second-degree atrioventricular block or escaped rhythm were not observed in all treatments (Table 2).

**Table 2:** Mean ± standard deviation of the cardiorespiratory variables in control (CONT), tramadol- lidocaine (TRM- LID), butrophanol-lidocaine (BUT-LID) treatments at MAC determination 2 min before applying noxious stimulus obtained from 10 female dogs in each treatment.

	**CONT**	**TRM-LID**	**BUT-LID**
**T (** **°** **C)**	37.8 ± 0.18	38.1 ± 0.23^*^	37.9 ± 0.13
**HR (beats minute** ^**−1**^ **)**	110.8 ± 14.6	91.5 ± 12.1^*^	106.4 ± 14.9
**MAP (mmHg)**	74.2 ± 12.4	75.9 ± 12.0	73.8 ± 13.8
**SpO**_**2**_ **(%)**	98.8 ± 0.9	98.0 ± 1.0^*^	98.7 ± 0.9
**P****e****′CO**_**2**_ **(mmHg)**	37.4 ± 1.6	37.9 ± 1.6	37.3 ± 1.5
***f*_R_** **(breaths minute**^**−1**^**)**	12	12	12

T, esophageal temperature; HR, heart rate; MAP, mean arterial pressure; SpO_2_, oxygen saturation using pulse oximetry; Pe′CO_2_, end-tidal carbon dioxide; f_*R*_, respiratory rate. ^*^Statistically different from CONT.

## Discussion

BUT–LID demonstrated a significant higher sparing effect than CONT. The cardiorespiratory variables in all treatments remained within the clinically acceptable range, although HR was maintained at a lower level in TRM–LID than in CONT and BUT–LID treatments.

The loading dose and CRI for the TRM, LID, and BUT treatments used in this study were based on those previously used in dogs (1, 11, 15). The MAC for the CONT group in the present study is similar to that observed in previous research (14, 19, 23, 24), in which the mean MAC value was 2.1–2.4%.

The sevoflurane MAC-sparing effects were (mean ± SD) 7.05 ± 22.20% and 19.90 ± 5.91% for TRM–LID and BUT–LID infusions, respectively. In a previous study, the observed sevoflurane MAC-sparing by TRM–LID was 30.1 ± 10.7% (23). It was observed a lower efficacy for the TRM–LID treatment in this group. This difference may be due to smaller dose of tramadol used in the current study (1.3 mg kg^−1^ h^−1^) while Thengchaisri and Mahidol (23) used larger dose of tramadol (2.6 mg kg^−1^ h^−1^). Many previous studies have illustrated different tramadol metabolism in different dog populations (25–27). These metabolism differences will lead to variable clinical efficacy in dogs (28). Also TRM–LID group need longer time for MAC evaluation (224 ± 50 min) due to differences in tramadol metabolism between dogs (23, 28). Although no previous studies examined the BUT–LID CRI sparing effect in dogs, but in one study (29) butorphanol was administered in bolus doses up to 0.8 mg kg^−1^ i.v. The authors did not demonstrate alterations in halothane MAC values. The sparing effect of BUT-LID treatment is significantly reduced in the current study when compared to CONT treatment. Previous studies demonstrated a significant decrease in enflurane MAC (15 ± 4%) after administration of 0.3 mg kg^−1^ i.v. butorphanol (30) and isoflurane MAC (20.26 ± 12.91%) after i.v. butorphanol 0.4 mg kg^−1^ (31). However, no significant difference in isoflurane concentration was detected between the CRI of fentanyl (potent analgesic) and the CRI of butorphanol during unilateral total mammary gland resection in dogs (19). We did not detect significant differences between the sparing effect of TRM–LID and BUT–LID treatments. According to our study, the sparing effect of TRM-LID and BUT-LID treatments did not differ significantly from one another, likely due to tramadol's low affinity for μ-opioid and κ-opioid receptors, exerting a weak agonist effect (32). In addition, butorphanol is a mild opioid drug that predominantly acts on the κ receptor and only partially on the μ receptor (19).

In the current study, BUT–LID provided the greatest reduction in required sevoflurane and MAC (1.9 ± 0.29), less than CONT (2.4 ± 0.36). The TRM–LID combination treatment resulted in a non-significant reduction in MAC value (2.1 ± 0.53). The study concluded that butorphanol's effect on MAC is superior than tramadol. The same results were illustrated previously as tramadol was associated with smaller changes in sedation score in dogs than morphine and butorphanol. This effect may be attributed to tramadol's low affinity for μ receptors (33). In dogs, butorphanol doses of 0.2 and 0.4 mg kg^−1^ were found to reduce the MAC by 15% (34) and 20% (31), respectively.

Using a multimodal analgesic approach such as the BUT–LID combination enhanced the sevoflurane MAC-sparing activity through a synergistic effect, the interaction of each analgesic's different mechanisms (20). We propose that the significant sparing ratio and reduction of sevoflurane requirements observed in this study may inform its use in clinical practice in canine, especially regarding to the undesired effect of excess inhalational anesthetic agents.

HR was slightly lower in the TRM–LID group than in the CONT and BUT–LID groups without affecting the mean arterial blood pressure. In a previous study, researchers observed a slightly lower heart rate in the tramadol infusion group (88 ± 8 bpm) and the tramadol–lidocaine infusion group (89 ± 6 bpm) than in the baseline (23). The moderate HR decreases we observed in the treatment groups compared to the control group seem to be due to reduced myocardial contractility. However, an increased vagal tone may result in bradycardia (35). Pulmonary depression, such as decreases in *f*_R_ and SPO_2_ and a rise in Pe′CO_2_, was not observed in BUT–LID treatments. This was similarly reported in dogs (33). Slight decrease in SPO_2_ was noticed in the TRM–LID than CONT and BUT–LID treatments, but it was still within clinical acceptable range (10, 36). This characteristic sign (no clinical respiratory depression) is considered one of the main advantages of using butorphanol over all other opioids.

The main limitations of the current study include no measurement of cardiovascular parameters such as invasive arterial pressure, SVR, and CO. Therefore, investigators could not comprehensively evaluate the cardiovascular effect of TRM–LID and BUT–LID co-infusion in dogs anesthetized with sevoflurane. However, it was clear that the BUT–LID co-infusion resulted in minimal cardiorespiratory changes with significant MAC reduction value and significant sparing effect in the dogs of this study. Furthermore, in the authors' experiment, they used clinically normal, healthy female dogs, which may not be the same as in male dogs or dogs with clinical problems. Another limitation is that the plasma concentrations of tramadol and butrophanol were not measured in the current study, so the pharmacodynamic effects of tramadol and butrophanol could not be detected. Researchers should keep these limitations in mind while designing future studies.

## Conclusion

This study demonstrated that BUT–LID co-infusion results in significant sparing effect of MAC requirements and a notable sevoflurane MAC reduction with minimal cardiorespiratory changes. It maintains MAP, SPO_2_, HR, Pe′CO_2_, *f*_R_ and esophageal temperature within clinically normal values for healthy mongrel female dogs. These results may inform improvements in dogs clinical practice. However, we did not detect any difference in MAC-sparing percentages between TRM–LID and BUT–LID treatments. Therefore, more studies are needed to confirm the present study's findings and to investigate cardiorespiratory parameters.

## Data availability statement

The raw data supporting the conclusions of this article will be made available by the authors, without undue reservation.

## Ethics statement

The animal study was reviewed and approved by the Ethics Committee of King Faisal University.

## Author contributions

MM: designed research, performed research, collected data, analyzed data, and wrote the paper. AA: performed research, collected data, and analyzed data. MK and HB: analyzed data and wrote the paper. WE-D: performed research and analyzed the data. SF: designed research, performed research, collected data, and analyzed data. All authors revised the paper and approved submission.
